# Tissue Distribution of Hirsutine and Hirsuteine in Mice by Ultrahigh-Performance Liquid Chromatography-Mass Spectrometry

**DOI:** 10.1155/2020/7204315

**Published:** 2020-04-21

**Authors:** Quan Zhou, Jianshe Ma, Limei Chen

**Affiliations:** ^1^The Laboratory of Clinical Pharmacy, The People's Hospital of Lishui, Lishui 323000, China; ^2^School of Basic Medicine, Wenzhou Medical University, Wenzhou 325035, China; ^3^Department of Anesthesiology, The First Affiliated Hospital of Wenzhou Medical University, Wenzhou 325000, China

## Abstract

Hirsutine and hirsuteine were two alkaloid monomers extracted from the traditional Chinese medicine *Uncaria rhynchophylla*, which have pharmacological effects such as antihypertension, anti-infection, and heart protection. An ultrahigh-performance liquid chromatography-mass spectrometry was established for the determination of hirsutine and hirsuteine in tissues (liver, kidney, heart, spleen, brain, and lung), and their absorption, distribution, and metabolism were studied for providing information on its pharmacological mechanism. UPLC BEH C18 column (2.1  mm × 100  mm, 1.7 *μ*m) was used for chromatographic separation. The mobile phase was acetonitrile-0.1% formic acid, with a gradient elution, and the total run time was 4 min. Electrospray was used in the positive ion mode, and the multiple reaction monitoring (MRM) mode was for quantification. The acetonitrile precipitation method was used to remove protein-treated mouse plasma and tissue homogenate samples. In the concentration range of 2–5000 ng/g, hirsutine and hirsuteine in tissues showed good linearity (*r* > 0.995), and the lower limit of quantification was 2 ng/g. In the plasma and liver tissues, the interday and intraday precision of hirsutine and hirsuteine was less than 15%, the accuracy was between 90.9% and 110.1%, and the average recovery was better than 73.0%. The matrix effect was between 86.2% and 104.7%. The results showed that the precision, accuracy, recovery, and matrix effects meet the requirements for the study on the distribution of hirsutine and hirsuteine. After intraperitoneal administration of 10 mg/kg hirsutine and hirsuteine in mice, the distribution levels were highest in liver and kidney tissues, followed by the spleen and lung. Hirsutine and hirsuteine were low in brain tissue, but had obvious distribution, suggesting that they may pass through the blood-brain barrier.

## 1. Introduction

*Uncaria rhynchophylla*（Miq.）Miq. ex Havil. is a commonly used Chinese medicine and belongs to the *Uncaria Schreber* nom. cons. [1–3]. Pharmacological studies show that *Uncaria rhynchophylla* has an antagonistic effect on Ca^2+^, which can block external Ca^2+^ influx and release of internal Ca^2+^ [4]. It has a strong inhibitory effect on the cardiovascular system and significantly reduces blood pressure; it has obvious nerve conduction block infiltration anesthesia and spinal canal anesthesia; it has a significant inhibitory effect on the synaptic transmission process of the central nervous system and has antiepileptic effects [5–7]. It can be used to treat hypertension, sedation, sleeping, and antispasmodic effects [8–12]. The indole alkaloids of *Uncaria rhynchophylla* are the main active ingredients.

The distribution of drugs in the body is closely related to the pharmacological effects of drugs, and their distribution determines the strength and duration of the effect [13–15]. It is still unclear whether hirsutine and hirsuteine can be absorbed into the blood and its metabolism and tissue distribution. However, studies on the tissue distribution of hirsutine and hirsuteine have not been reported in the literature.

Tissue distribution is an indispensable part of pharmacokinetic research, and it can clarify the change trend of tissue drug concentration with time of administration, and then evaluate the targeting and tissue accumulation of the drug in the body, and judge the safety and effectiveness of the drug [16–18]. In this paper, we established a UPLC-MS/MS method for the quantitative determination of hirsutine and hirsuteine in tissue homogenates.

## 2. Experimental

### 2.1. Reagents and Animals

Hirsutine and hirsuteine (purity >98%) and diazepam (internal standard, purity >98%) were purchased from Chengdu Munster Pharmaceutical Co., Ltd (Chengdu, China). Chromatographically pure acetonitrile, methanol, and formic acid were purchased from Tedia (Ohio, USA). Ultrapure water was prepared by a Millipore purification system (Bedford, MA, USA). ICR mice (20 ± 2g, male) were provided by the Animal Experiment Center of Wenzhou Medical University.

### 2.2. Instruments and Conditions

ACQUITY H-Class UPLC and XEVO TQS-micro Triple Quadrupole Mass Spectrometer (Waters Corp, Milford, MA, USA). Masslynx 4.1 software (Waters Corp.) was used for data acquisition and instrument control.

The chromatographic column was Waters UPLC BEH C18 (2.1 mm × 50 mm, 1.7 *μ*m), and the column temperature was 40°C. The mobile phase consisted of 0.1% formic acid-acetonitrile, and the flow rate was 0.4 mL/min. The gradient elution procedure was: 0–0.2 min, acetonitrile 10%; 0.2–1.5 min, acetonitrile 10%–80%; 1.5–2.0 min, acetonitrile 80%; 2.0–2.5 min, acetonitrile 80%–10%; and 2.5–4.0 min, 10% acetonitrile.

The mass spectrometry parameters were set to a capillary voltage of 2.5 kV, a source temperature of 150°C, a desolvation temperature of 500°C, and nitrogen as the desolvation gas (900 L/h) and cone gas (50 L/h). The MRM mode was used to quantitatively analyze hirsutine m/*z* 369.2⟶226.0, hirsuteine m/*z* 367⟶169.9, and internal standard (diazepam) m/*z* 285.1⟶193.3.

### 2.3. Preparation of Reference Solution

Hirsutine and hirsuteine were accurately weighed into 10 mL volumetric flasks, and hirsutine and hirsuteine (1.0 mg/mL) stock solution was prepared with methanol-water (50 : 50). Diazepam was accurately weighed into a 10 mL volumetric flask, and a diazepam (1.0 mg/mL) stock solution was prepared with methanol-water (50 : 50). The stock solution was diluted with methanol to a working solution of various concentrations, stored in a refrigerator at 4° C, and kept at room temperature during use.

### 2.4. Sample Processing

Tissue samples are taken out of the refrigerator and thawed. The tissue is ground and then weighed (50 mg). The weighed tissue was placed in a 1.5 mL centrifuge tube, and 200 *μ*L of internal standard (50 ng/mL) acetonitrile was added. After vortexing for 1.0 min, it is placed in a low-temperature high-speed centrifuge (13000 r/mim, 4°C) for 10 min. Then, 150 *μ*L of the supernatant is taken and placed it in a cannula into a sample bottle, and 2 *μ*L is taken for injection for UPLC-MS/MS detection.

### 2.5. Standard Curve

Proper amount of hirsutine and hirsuteine working solution is added to blank tissue (brain, kidney, heart, liver, spleen, and lung) to prepare hirsutine and hirsuteine standard curve. The concentration is in the range of 2–5000 ng/g, and the concentrations are 2, 5, 20, 100, 200, 500, 1000, 2000, and 5000 ng/g. In the same way as the standard curve, quality control samples (QC) were prepared at three concentrations (4, 450, and 4500 ng/g).

### 2.6. Method Validation

#### 2.6.1. Selectivity

For the analysis of blank tissue and blank tissue spiked with hirsutine, hirsuteine, and internal standard, tissue samples were administered intraperitoneally at 10 mg/kg in mice to evaluate the selectivity of the method.

#### 2.6.2. Linear

Standard working solutions were prepared into different standard series with standard concentrations ranging from 2 to 5000 ng/g. Under the same conditions as the tissue samples to be measured, the peak area of each peak is measured, and the ratio of the peak area to the internal standard peak area is used to draw a standard curve for the sample concentration.

#### 2.6.3. Precision and Accuracy

Precision and accuracy were assessed by measuring tissue samples of these three QC sample concentration levels (4, 450, and 4500 ng/g) in 6 replicates. Intra- and interday precision is determined by measuring QC samples at three concentration levels for three consecutive days. The intraday and interday accuracy were determined by measuring the consistency of the average value of the QC samples at three concentration levels with the true value for three consecutive days.

#### 2.6.4. Recovery and Matrix Effects

The recovery was evaluated by comparing the measured peak areas of low, medium, and high concentration QC samples with the corresponding standard peak areas. The peak areas obtained by preparing blank plasma or tissue after sample processing and adding standard solutions to prepare low, medium, and high concentrations (4, 450, and 4500 ng/g) were compared with the corresponding standard peak areas by using acetonitrile-0.1% formic acid (1 : 1, v/v) to assess matrix effects.

#### 2.6.5. Stability

The mouse QC samples were analyzed at low, medium, and high concentrations (4, 450, and 4500 ng/g) by placing them in three storage conditions to investigate trichostine and dehydropilus in mouse plasma or tissue, respectively. Stability in injection vials, short-term stability (2 hours at room temperature), long-term stability (−20°C, 30 days), freeze-thaw stability (−20°C to room temperature), and 3 concentrations of freshly prepared (4, 450, and 4500 ng/g) standard samples were compared for peak area to investigate stability.

### 2.7. Tissue Distribution

Thirty mice were injected intraperitoneally with 10 mg/kg hirsutine and hirsuteine, and then at 0.25, 0.5, 2, 4, 6, and 12 h, with 4% chloride, 5 mice were sacrificed after being subjected to aldehyde anesthesia, and tissue samples were collected (liver, kidney, heart, spleen, brain, and lung). After collection, the surface was washed with ice-cold saline and dried with filter paper. Tissue samples were then stored at −20°C.

## 3. Results and Discussion

### 3.1. Method Optimization

Electrospray ESI positive and negative electrode selection was often evaluated in methodological studies [19]. In this paper, MRM positive ion mode was used for quantitative analysis. By optimizing ion ionization parameters, the selected quantified ions were set at m/*z* 369.2⟶226.0 for hirsutine, and m/*z* 367⟶169.9 for hirsuteine.

HPLC conditions were as far as possible from separating endogenous interfering substances from analyte and internal standard, and the chromatographic behavior of the column and mobile phase plays a decisive role [20–22]. BEH C18 (2.1 mm × 50 mm, 1.7 *μ*m) can obtain satisfactory peak time and peak shape. We also tried different mobile phases, such as acetonitrile-water, methanol-water, acetonitrile-0.1% formic acid, and methanol-0.1% formic acid, and found that the peak shape and sensitivity of acetonitrile-0.1% formic acid were the best. Therefore, a BEH C18 (2.1 mm × 50 mm, 1.7 *μ*m) column was used in this work, and acetonitrile-0.1% formic acid was used.

Prior to UPLC-MS/MS analysis, removal of proteins and potential interference was a key point in method development [23–25]. In our previous work, we found that the fast and simple acetonitrile precipitation method has the best effect on rat plasma. Considering that the tissue sample was more complex than plasma, 50 mg of tissue homogenate was processed by 200 *μ*L acetonitrile precipitation. As a result, it was found that the direct protein precipitation method can obtain acceptable extraction recovery and matrix effect.

### 3.2. Method Validation

#### 3.2.1. Selectivity

**Figure 1:**
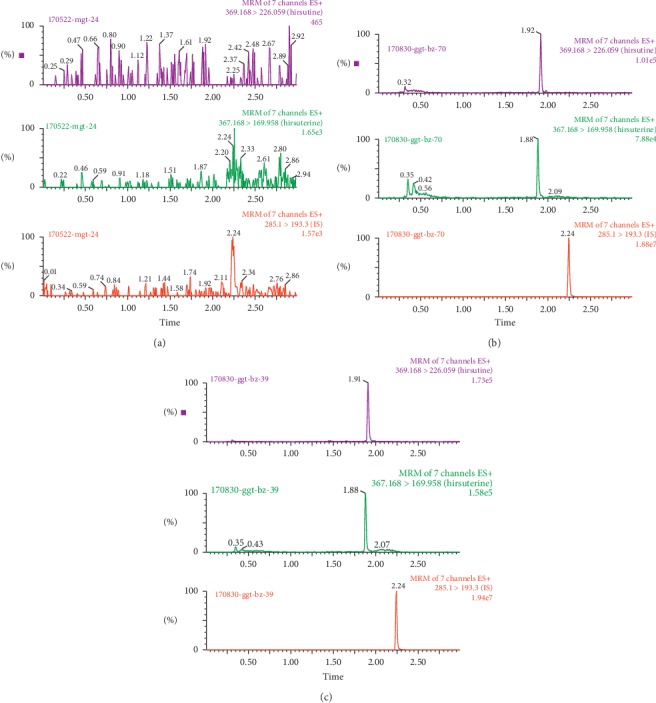
UPLC-MS/MS spectra of hirsutine, hirsuteine, and internal standard in mouse liver. (a) Blank tissue. (b) Blank liver spiked into hirsutine, hirsuteine, and internal standard. (c) A liver sample of mice injected intraperitoneally with 10 mg/kg of hirsutine and hirsuteine.

Figure 1 shows typical UPLC-MS/MS spectra of blank liver tissue, blank liver tissue with hirsutine, hirsuteine, and internal standard, and a mouse sample. No interfering substances were found at the retention times of hirsutine, hirsuteine, and internal standard.

#### 3.2.2. Standard Curve and Sensitivity

In the concentration range of 2–5000 ng/g, the peak area ratio and concentration linearly regress. The linear equations of various tissues are shown in Table 1. The lower limit of quantification of tissues is 2 ng/g, the precision is between 10% and 18%, and the accuracy is between 85% and 118%. The detection limit is 0.5 ng/g, and the signal-to-noise ratio is 3.

#### 3.2.3. Precision, Accuracy, Recovery, and Matrix Effects

The data in Table 2 show that, for hirsutine and hirsuteine in tissues, the intraday precision is less than 13.5%, and the interday precision is less than 14.5%. The intraday accuracy is between 90.8% and 110.1%, and the interday accuracy is between 88.8% and 113.6%. The average recovery is between 73.0% and 95.5%, and the matrix effect is between 86.2% and 104.7%. The results show that the precision, accuracy, recovery, and matrix effects meet the requirements for the study on the distribution of hirsutine and hirsuteine.

#### 3.2.4. Stability

Mouse tissue samples were tested at room temperature for 2 hours, at −20°C for 30 days, and freeze-thaw stability tests were also conducted. The results showed that the variation of hirsutine and hirsuteine was within ±15%, and RSD was within 15%, indicating that they were stable.

### 3.3. Tissue Distribution

**Figure 2:**
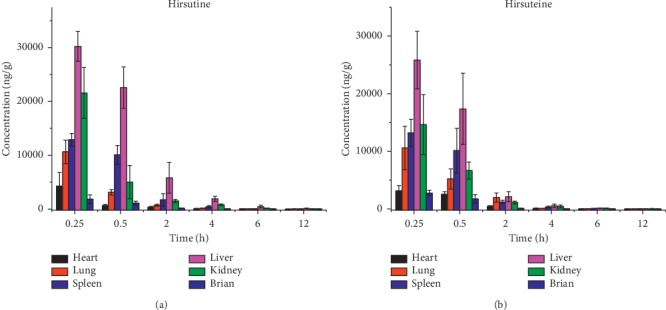
Tissue distribution in mice after intraperitoneal administration of 10 mg/kg of hirsutine and hirsuteine.

After intraperitoneal injection of 10 mg/kg hirsutine and hirsuteine to mice, the mean time distribution curve of tissues are shown in Figure 2. Tissue samples with concentrations above 5000 ng/g were diluted 10-fold with blank tissue.

Although some literature studies have reported the pharmacokinetics of hirsutine or hirsuteine in rats [26–29], no literature has reported the tissue distribution of hirsutine or hirsuteine. Han et al. established a UPLC-MS/MS method to determine hirsutine and hirsuteine in rat plasma, used 100 *μ*L plasma, and needed 4 min for one sample [26]. Wu et al. used the UPLC-MS/MS method to determine rhynchophylline and hirsutine in plasma after oral administration; one sample needed 6 min for analysis and liquid-liquid extraction using 100 *μ*L plasma [27]. Kushida et al. developed a LC/MS-MS method for the simultaneous determination of seven alkaloids in rat plasma and brain within 24 min, using 100 *μ*L plasma or brain [29]. However, the tissue distribution of hirsutine and hirsuteine in rats or mice was not reported in these literature studies.

It was rapidly distributed in various tissues and organs through blood circulation, and the highest level was distributed in liver and kidney tissues, followed by spleen and lung. This phenomenon indicates that it was widely distributed in mice, which may be related to the higher lipid solubility of hirsutine and hirsuteine. With the extension of the administration time, the drug concentrations of hirsutine and hirsuteine in each tissue decreased rapidly, and the concentration of each tissue was already low at 6 h. The highest concentration is in liver and kidney tissues. Metabolism of hirsutine and hirsuteine mainly occurs in liver tissues, which were excreted in renal tissues, and may be related to large blood flow and good circulation in liver and kidney tissues. Hirsutine and hirsuteine are low in brain tissue but have obvious distribution, suggesting that the drug may pass through the blood-brain barrier.

## 4. Conclusion

We established a method based on UPLC-MS/MS for the detection of hirsutine and hirsuteine in tissues, with a linear range of 2–5000 ng/g. The UPLC-MS/MS was simple and sensitive, and 50 mg of tissue was required. After mice were injected intraperitoneally with 10 mg/kg hirsutine and hirsuteine, the distribution levels were highest in liver and kidney tissues, followed by spleen and lung. The results of this study also help to better understand the pharmacological mechanisms of hirsutine and hirsuteine.

## Figures and Tables

**Table 1:** Linear equations, correlation coefficients, lower limit of quantification, and detection limit of hirsutine and hirsuteine in mouse tissue.

Compound	Name	Linear equations	Concentration range (ng/mL)	Correlation coefficient	Lloq	Lod
Hirsutine	Kidney	*y* = 0.0008*x* + 0.0006	2–5000	0.9983	2	0.05
	Liver	*y* = 0.0007*x* + 0.0003	2–5000	0.9982	2	0.05
	Heart	*y* = 0.0009*x* + 0.0002	2–5000	0.9967	2	0.05
	Spleen	*y* = 0.0008*x* + 0.0005	2–5000	0.9981	2	0.05
	Brain	*y* = 0.0005*x* + 0.0004	2–5000	0.9952	2	0.05
	Lung	*y* = 0.0004*x* + 0.0002	2–5000	0.9971	2	0.05

Hirsuteine	Kidney	*y* = 0.0008*x* + 0.0007	2–5000	0.9977	2	0.05
	Liver	*y* = 0.0006*x* + 0.0004	2–5000	0.9984	2	0.05
	Heart	*y* = 0.0007*x* + 0.0004	2–5000	0.9973	2	0.05
	Spleen	*y* = 0.0009x - 0.0001	2–5000	0.9991	2	0.05
	Brain	*y* = 0.0006x - 0.0002	2–5000	0.9975	2	0.05
	Lung	*y* = 0.0004*x* + 0.0002	2–5000	0.9969	2	0.05

**Table 2:** Precision, accuracy, matrix effect, and recovery of hirsutine and hirsuteine in mouse tissue (*n* = 6).

Compound	Tissue	Concentration (ng/mL)	Precision (%)		Accuracy(%)		Recovery	Matrix effect
			Intraday	Interday	Intraday	Interday		
Hirsutine	Kidney	4	8.4	7.4	96.5	103.3	76.3	91.7
		450	3.0	5.3	98.6	98.2	73.0	86.2
		4500	8.2	4.5	101.9	96.8	77.6	86.5
	Liver	4	12.9	14.5	97.1	102.9	85.9	94.9
		450	10.2	10.6	109.1	90.9	85.9	91.0
		4500	4.0	8.0	92.8	107.2	77.8	94.0
	Heart	4	12.4	13.3	90.8	89.4	85.9	95.2
		450	7.8	5.5	104.7	104.2	79.3	95.1
		4500	2.6	9.9	98.2	101.8	89.3	96.1
	Spleen	4	7.0	11.2	105.0	96.1	89.3	98.4
		450	8.7	13.2	96.1	96.5	77.3	91.1
		4500	2.6	4.2	101.0	103.2	80.2	97.1
	Brain	4	5.4	3.5	98.2	103.3	82.6	93.7
		450	3.7	2.8	104.3	102.5	84.5	99.2
		4500	2.9	2.9	97.0	98.8	93.7	98.2
	Lung	4	8.5	8.5	100.2	110.0	91.8	96.5
		450	3.6	5.2	106.9	94.1	92.8	98.6
		4500	3.3	4.5	94.6	108.1	95.5	101.9

Hirsuteine	Kidney	4	9.0	7.8	96.2	103.1	76.5	92.5
		450	1.7	4.7	98.6	98.4	86.9	87.3
		4500	6.7	4.8	102.9	98.5	81.4	90.8
	Liver	4	11.6	12.2	96.5	113.6	86.9	102.5
		450	13.5	11.0	110.1	93.3	87.4	104.7
		4500	7.6	11.5	100.8	103.7	84.7	99.6
	Heart	4	9.6	13.0	93.8	88.8	83.6	103.4
		450	8.7	8.7	99.9	104.7	83.0	101.6
		4500	8.7	7.4	101.8	99.5	87.4	103.8
	Spleen	4	10.5	12.9	94.1	93.6	83.5	97.6
		450	7.9	8.7	107.7	95.3	73.9	95.4
		4500	8.6	3.2	99.3	103.6	85.9	103.9
	Brain	4	12.8	10.6	107.0	109.9	88.5	102.2
		450	8.3	8.3	103.8	98.6	88.3	95.6
		4500	4.2	7.2	98.8	98.3	85.6	104.5
	Lung	4	11.2	13.8	104.5	94.3	83.4	97.3
		450	6.2	7.3	96.9	104.8	80.2	103.0
		4500	3.4	8.3	99.6	98.3	86.9	95.9

## Data Availability

The data used to support the findings of this study are included within the article.

## References

[1] Geng C.-A., Yang T.-H., Huang X.-Y., Ma Y.-B., Zhang X.-M., Chen J.-J. (2019). Antidepressant potential of Uncaria rhynchophylla and its active flavanol, catechin, targeting melatonin receptors. *Journal of Ethnopharmacology*.

[2] Wang Y.-L., Dong P.-P., Liang J.-H. (2018). Phytochemical constituents from Uncaria rhynchophylla in human carboxylesterase 2 inhibition: kinetics and interaction mechanism merged with docking simulations. *Phytomedicine*.

[3] Shin S. J., Jeong Y., Jeon S. G. (2018). Uncaria rhynchophylla ameliorates amyloid beta deposition and amyloid beta-mediated pathology in 5XFAD mice. *Neurochemistry International*.

[4] Ndagijimana A., Wang X., Pan G., Zhang F., Feng H., Olaleye O. (2013). A review on indole alkaloids isolated from Uncaria rhynchophylla and their pharmacological studies. *Fitoterapia*.

[5] Wang X., Qiao Z., Liu J., Zheng M., Liu W., Wu C. (2018). Stereoselective in vitro metabolism of rhynchophylline and isorhynchophylline epimers of Uncaria rhynchophylla in rat liver microsomes. *Xenobiotica*.

[6] Li T., Xu K., Che D., Huang Z., Jahan N., Wang S. (2018). Endothelium-independent vasodilator effect of isocorynoxeine in vitro isolated from the hook of Uncaria rhynchophylla (Miquel). *Naunyn-Schmiedeberg’s Archives of Pharmacology*.

[7] Lan Y. L., Zhou J.-J., Liu J. (2018). Uncaria rhynchophylla ameliorates Parkinson’s disease by inhibiting HSP90 expression: insights from quantitative proteomics. *Cellular Physiology and Biochemistry*.

[8] Guo Q., Yang H., Liu X. (2018). New zwitterionic monoterpene indole alkaloids from Uncaria rhynchophylla. *Fitoterapia*.

[9] Chen X. W., Yang Z. D., Sun J. H., Song T. T., Zhu B. Y., Zhao J. W. (2018). Colletotrichine A, a new sesquiterpenoid from Colletotrichum gloeosporioides GT-7, a fungal endophyte of Uncaria rhynchophylla. *Natural Product Research*.

[10] Wei X., Jiang L. P., Guo Y. (2017). Indole alkaloids inhibiting neural stem cell from Uncaria rhynchophylla. *Natural Products and Bioprospecting*.

[11] Loh Y. C., Ch’ng Y. S., Tan C. S., Ahmad M., Asmawi M. Z., Yam M. F. (2017). Mechanisms of action of Uncaria rhynchophylla ethanolic extract for its vasodilatory effects. *Journal of Medicinal Food*.

[12] Hishiki T., Kato F., Tajima S. (2017). Hirsutine, an indole alkaloid of Uncaria rhynchophylla, inhibits late step in dengue virus lifecycle. *Frontiers in Microbiology*.

[13] Ye W. J., Lin C. L., Lin G. Y. (2019). Tissue distribution of engeletin in mice by UPLC-MS/MS. *Current Pharmaceutical Analysis*.

[14] Li T. R., Ye W. J., Huang B. G. (2019). Determination and pharmacokinetic study of echinatin by UPLC-MS/MS in rat plasma. *Journal of Pharmaceutical and Biomedical Analysis*.

[15] Wang X. Q., Wang S. H., Lin F. Y. (2015). Pharmacokinetics and tissue distribution model of cabozantinib in rat determined by UPLC-MS/MS. *Journal of Chromatography B-Analytical Technologies in the Biomedical and Life Sciences*.

[16] Gao X., Tsai R. Y. L., Ma J. (2020). Determination and validation of mycophenolic acid by a UPLC-MS/MS method: applications to pharmacokinetics and tongue tissue distribution studies in rats. *Journal of Chromatography B*.

[17] Hsiao C. H., Zhao J., Gao S. (2019). Development and validation of a rapid and sensitive UPLC-MS/MS assay for simultaneous quantification of paclitaxel and cyclopamine in mouse whole blood and tissue samples. *Biomedical Chromatography*.

[18] Zhang C., Ma W., Zhang Y. (2018). Pharmacokinetics, bioavailability, and tissue distribution study of angoroside C and its metabolite ferulic acid in rat using UPLC-MS/MS. *Front Pharmacology*.

[19] Li J., Jin Y., Fu H., Huang Y., Wang X., Zhou Y. (2019). Pharmacokinetics and bioavailability of gelsenicine in mice by UPLC-MS/MS. *Biomed Chromatography*.

[20] Luo Y., Li L. Y., Cai J. Z. (2019). Determination of RKI-1447 in rat plasma by UPLC-MS/MS and investigation on its pharmacokinetics, an effective ROCK1 and ROCK2 inhibitor. *Acta Chromatographica*.

[21] Song H. C., Huang Y. W., Zhu D. Q. (2019). Pharmacokinetic study of deltaline in mouse blood based on UPLC-MS/MS. *Current Pharmaceutical Analysis*.

[22] Weng Q. H., Weng T. T., Lin Y. J. (2019). Determination of buddleoside in rat plasma by UPLC-MS/MS and its pharmacokinetics. *Latin American Journal of Pharmacy*.

[23] Wang H. Y., Chen Y. C., Wan J. F. (2018). Determination of fargesin in rat plasma by UPLC-MS/MS and its pharmacokinetics application. *Latin American Journal of Pharmacy*.

[24] Hu L. M., Zhang M. M., Qu M. X. (2015). Determination of tuberostemonin in rat plasma by UPLC-MS/MS and its application to pharmacokinetic study. *Latin American Journal of Pharmacy*.

[25] Wang S. H., Ding T., Chen J. M. (2015). Development of a UPLC-MS/MS method for determination of tacrolimus and cyclosporine A in human whole blood. *Latin American Journal of Pharmacy*.

[26] Han A. X., Lin G. Y., Cai J. Z. (2019). Pharmacokinetic study on hirsutine and hirsuteine in rats using UPLC-MS/MS. *Acta Chromatographica*.

[27] Wu Y. T., Lin L. C., Tsai T. H. (2014). Determination of rhynchophylline and hirsutine in rat plasma by UPLC-MS/MS after oral administration of Uncaria rhynchophylla extract. *Biomedical Chromatography*.

[28] Nakazawa T., Banba K. I., Hata K., Nihei Y., Hoshikawa A., Ohsawa K. (2006). Metabolites of Hirsuteine and Hirsutine, the major indole alkaloids of Uncaria rhynchophylla, in rats. *Biological & Pharmaceutical Bulletin*.

[29] Kushida H., Fukutake M., Tabuchi M. (2013). Simultaneous quantitative analyses of indole and oxindole alkaloids of Uncaria Hook in rat plasma and brain after oral administration of the traditional Japanese medicine Yokukansan using high-performance liquid chromatography with tandem mass spectrometry. *Biomed Chromatography*.

